# An Autonomous Underwater Recorder Based on a Single Board Computer

**DOI:** 10.1371/journal.pone.0130297

**Published:** 2015-06-15

**Authors:** Manuel Caldas-Morgan, Alexander Alvarez-Rosario, Linilson Rodrigues Padovese

**Affiliations:** Department of Mechanical Engineering, University of São Paulo, São Paulo, SP, Brazil; Pacific Northwest National Laboratory, UNITED STATES

## Abstract

As industrial activities continue to grow on the Brazilian coast, underwater sound measurements are becoming of great scientific importance as they are essential to evaluate the impact of these activities on local ecosystems. In this context, the use of commercial underwater recorders is not always the most feasible alternative, due to their high cost and lack of flexibility. Design and construction of more affordable alternatives from scratch can become complex because it requires profound knowledge in areas such as electronics and low-level programming. With the aim of providing a solution; a well succeeded model of a highly flexible, low-cost alternative to commercial recorders was built based on a Raspberry Pi single board computer. A properly working prototype was assembled and it demonstrated adequate performance levels in all tested situations. The prototype was equipped with a power management module which was thoroughly evaluated. It is estimated that it will allow for great battery savings on long-term scheduled recordings. The underwater recording device was successfully deployed at selected locations along the Brazilian coast, where it adequately recorded animal and manmade acoustic events, among others. Although power consumption may not be as efficient as that of commercial and/or micro-processed solutions, the advantage offered by the proposed device is its high customizability, lower development time and inherently, its cost.

## Introduction

Underwater acoustic research has acquired great importance in recent years, either in monitoring man-generated acoustic events or in assessing its impact on marine life. When long-term research activities are to be executed around remote locations where manned missions are difficult, this kind of monitoring can be logistically and economically daunting.

Since the water medium is a very efficient sound transmitter, underwater acoustic studies are suitable in conducting cetacean ecology research given that it allows the detection of acoustic events occurring as far as hundreds of kilometers away [1]. Typically; commercial Passive Acoustic Monitoring (PAM) devices, such as [2–5], often use hydrophones in conjunction with adequate hardware with the purpose of transmitting and storing acoustic events. In order to perform this task reliably; these devices are robustly engineered, they commonly use proprietary software (source-code not available to the public) and are not very flexible (impossible or very difficult to modify the hardware). Usually, their price range is in the order of thousands of dollars [4]. Despite the different manufacturer features (such as sample rates, maximum allowable depth, materials and storage capacity among others), autonomous underwater recorders commonly consist of at least one hydrophone; located on the external surface of a waterproof enclosure. The hydrophone output passes through a signal-conditioning board before entering into an analog to digital converter (ADC). Subsequently, data is saved on some sort of storage device (HDD, Flash drive, SD card). Usually, these systems are powered by a set of batteries, commonly located within the same enclosure as the electronics. These set of batteries have configurations that often vary from manufacturer to manufacturer. Even though it is difficult to establish a direct comparison between manufacturers using the information available in the market, it was estimated that the power consumption of the aforementioned recorders might lie somewhere between 450 mW to 720 mW.

In order to overcome the involved costs or to find alternative custom solutions for specific working conditions, several research groups around the world have developed their own versions of autonomous underwater recorders. Besides battery life, one of the most challenging aspects of building such a recorder is related to the electronics that handle the analog to digital data conversion and storage. Some authors [6–8] have proposed solutions based on microprocessors/microcontrollers that can be very energy-efficient. Others have favored ultra-high storage and high autonomy at the expense of energy-efficiency [9]. Nonetheless, the development of such systems requires a profound knowledge in both electronics and programming that might be well beyond the knowledge of non-engineering oriented users. An initiative to build an underwater autonomous recorder using a single board computer was previously attempted in [10]; however, besides the resolution being inferior to 16 bit (data was sampled by a 12 bit PCM-3718HG data acquisition module), the autonomy of about 4.5 hours appeared to be low given that the device was powered by a 35.2 A·h 16V lithium battery. There has been even a simpler, yet interesting initiative, in which the recorder was based on a commercial MP3 player running open source firmware [11]. A more comprehensive list composed of more than 30 solutions for underwater audio recording devices (available as of 2013), especially aimed at the studying and monitoring of marine mammals, can be found in [12].

The motivation of this work was to produce an autonomous underwater recording device for scientific research; with the intention of finding equilibrium between complexity, functionality, processing capabilities, power consumption, low-cost and quality. This device was based on the Raspberry Pi, a single board computer developed by the Raspberry Pi foundation in the United Kingdom; with educational purposes [13]. Due to its low-cost and great flexibility, this computer has become increasingly popular among scientists and hobbyists. Besides having built-in memory, a microprocessor, USB ports and being able to run a Linux based operating system; it is equipped with a set of general purpose input/output ports which further increase the interaction with several kinds of electronic sensors.

The proposed device was able to digitalize underwater acoustic signals coming from a hydrophone and routed through a custom built signal-conditioning board. The digitalization was handled by a commercial USB audio codec connected to a Raspberry Pi Model A. The device was powered by a set of batteries located inside the same containing enclosure of the electronics. After testing extensively, both the mechanical resistance of the containing enclosure and the power consumption of the electronics, the device was deployed at different locations along the eastern and southeastern coast of Brazil, in missions related to cetacean research and marine traffic monitoring.

## Materials and Methods

Before describing the procedures used for the development and testing of the device under laboratory conditions; it is worth mentioning that the field activities related to the testing of the device took place in areas considered of public access located in the eastern and southeastern coast of Brazil. According to the Brazilian law, no specific permissions are required as long as there is no transgression of areas that are considered private, located within conservation units or that are of any military importance. These field activities did not involve direct interaction with any endangered or protected species.

**Fig 1:**
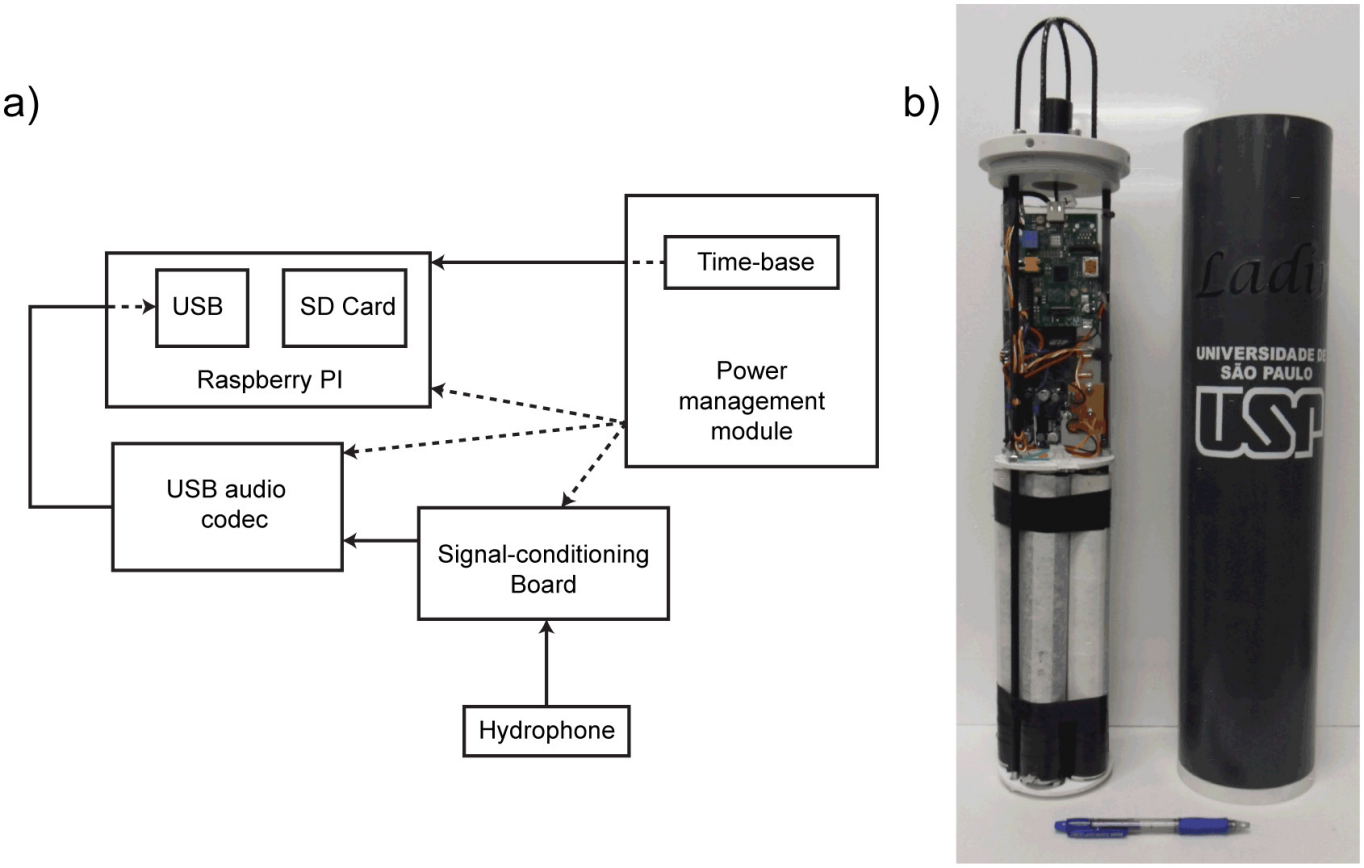
(a) The schematic representation shows the components of the autonomous underwater recording device and (b) the waterproof PVC enclosure, which contained the components.

The main components of the proposed autonomous underwater recorder are illustrated in Fig 1a. Besides the basic hardware components depicted; namely the hydrophone, a signal-conditioning board, the USB audio codec and the Raspberry Pi, there is a key set of components grouped inside the power management module (explained further ahead) that are related to a clock used to provide a time-base. With the exception of both the hydrophone and its stainless steel protection cage (which are mounted on the outer top surface), all of the hardware components were located inside a 50 cm long cylindrical PVC enclosure, having 114.30 mm of diameter and 9.52 mm of wall thickness (Fig 1b). This enclosure was tested inside a pressure chamber up to a gauge pressure of 10 bar (approximately 99.5 m of water depth), without showing signs of neither mechanical failure nor leakage.

### Hydrophone and signal-conditioning

**Fig 2:**
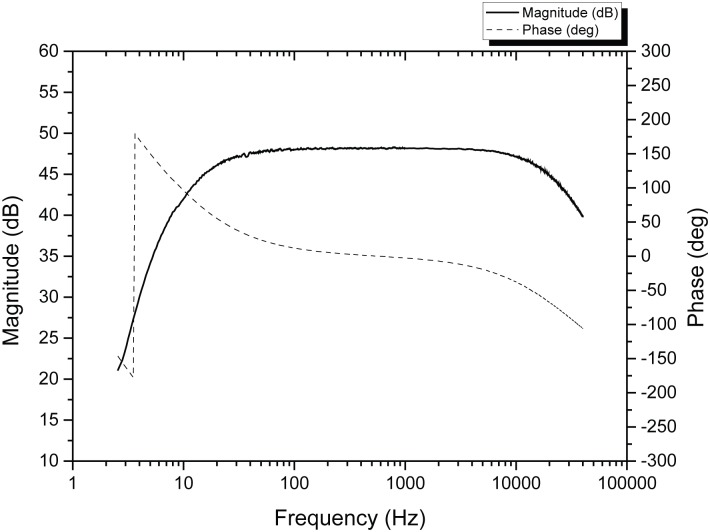
Experimental frequency response obtained with a particular tuning of the signal-conditioning board.

In principle, any kind of hydrophone can be used with the proposed device; from home-made models such as [14] to professional ones having superior specifications, such as the commercial model advertised in [15]. Notwithstanding that; the results presented in the this work were obtained with a custom-made hydrophone consisting of a polyurethane sealed PZT-5 piezoelectric cylinder (25 mm of diameter, 32 mm of length after embedding), with omnidirectional sensitivity of -198 dB re: 1 V/μPa and flat frequency response from DC up to 30 kHz within +/-2 dB. This hydrophone was connected to a custom-built signal-conditioning board. This board consisted of two cascaded non-inverting band pass filters with individual tunable gains and bandwidth. For the recordings mentioned further ahead in this work, the gain of the stages was set at 28 dB and 20 dB respectively. The combined gain of 48 dB was experimentally found to be well suited to the conditions at the selected deployment sites. This cascaded approach was chosen in order to preserve the gain-bandwidth product in circumstances where very high levels of gain are needed. Additionally, by using this configuration, it was possible to provide the signal-conditioning board with separate outputs coming from the first and the combined stages respectively. This feature could enable simultaneous two channel (stereo) recordings to be made at different gains using a single hydrophone; however, all the signals presented in this work were acquired using only the combined stage output. Fig 2 Illustrates the magnitude and phase response of a signal-conditioning board tuned to have a total gain of 48dB with a -3 dB bandwidth from 15 Hz to 20 kHz.

### USB audio codec

After being filtered and amplified, the signal was routed into one of the line-in inputs of a Burr Brown PCM2902 audio codec (found in consumer electronics such as the Behringer UCA222 Audio Interface) connected to the USB port of the Raspberry Pi. This audio codec has an internal 16 bit Delta-Sigma ADC with hardware defined sampling rates of 8, 11.025, 16, 22.050, 32, 44.100 and 48 kHz [16] that allows the voltage coming from the signal-conditioning board to be digitalized and stored.

The vertical scale of the digitalized files (i.e. PCM WAV files) is known as Full Scale (Fs). Normally, when audio files are open in a computer, using any suitable software, the amplitude of the recorded waveform is represented by 2^16^ Fs digital levels normalized between -1 and 1. Thus, a calibration procedure was needed in order to establish a relationship between these Fs levels and the voltage that produced them. This procedure involved the recording of a known pattern, which was made at a sample rate of 48 kHz in PCM WAV format. Preliminary tests showed that input waveforms with peak amplitudes as high as 2.25 V produced saturation in the recorded WAV file. Subsequently, a compatible calibration pattern was produced using an Agilent 33250A signal generator. This pattern was obtained by modulating a sine wave of 1 kHz and 2.25 V of peak amplitude with a crescent ramp function of 1 Hz of frequency. The rate of change of the amplitude of the Fs values was calculated by analyzing the obtained recording. As the rate of change of the peak input amplitude was known, the calibration constant could be found using the proportion between the two rates of change.

The use of a modulated signal of 1 kHz of frequency was judged adequate given that when sampling at 48 kHz, the digital filters of the ADC inside the USB audio codec provide a -3dB bandwidth between 3.5 Hz and 23.75 kHz [16].

The calibration results, in conjunction with the sensitivity of the hydrophone, allowed the acquired audio waveforms to be transformed into sound pressure level (SPL) variations relative to time; and accordingly, to gain a physical meaning. This was necessary in order to calculate the self-noise of the device, which was done by programming it to make a continuous recording (at 48 kHz) before positioning it inside an anechoic chamber. This chamber was supported by rubber feet, which helped to decouple it from low frequency vibrations. Eventually, the electric power supply of the testing facility was interrupted in order to avoid any possible electromagnetic interference. The self-noise of the device was estimated by calculating the RMS value of the resulting recording and expressing it in dB re: 1 V/μPa. Analogously, the background noise at the deployment sites was calculated by analyzing the quieter intervals registered during long-time recordings.

### Single board computer

The USB audio codec was hosted by a Raspberry Pi Model A; a single board computer based on a BCM2835 system on a chip (SoC) with 256 MB of RAM, one USB port and no Ethernet port. This computer also features a 26 pin port, where 17 of those pins are GPIO (general purpose input/output), that allows for communication using protocols such as I^2^C, SPI and serial [17]. The Raspberry Pi can be powered supplying 3.3 V and 5 V to pins 1 and 2 respectively; however, it is commonly powered just by 5 V as the board has an integrated 3.3 V linear dropout voltage regulator internally connected to pin 1. The Raspberry Pi is capable of running Linux, an open-source operating system that allows the user to perform some moderate to difficult tasks through a set of simple command lines based on routines (shell-scripts). The Linux distribution chosen to work with was Arch Linux, which is specifically designed for systems based on ARM technology. As this distribution lacks the drivers needed to interact with the USB audio codec, it was necessary to install the Advanced Linux Sound Architecture (ALSA) package in order to enable audio support. After having the adequate drivers installed, the next step was to install the SoX (Sound eXchange) package. This piece of software was used to configure the recording settings (i.e. sample rate, number of channels, bit depth), to choose the file format (between RAW binary or PCM WAV), to perform further operations such as splitting the files during long-term recordings (which, in the device, was configured to occur every 300 s by default) as well as filtering and resampling as needed. The complete list of SoX commands, with further information regarding its capabilities, is detailed in [18].

**Fig 3:**
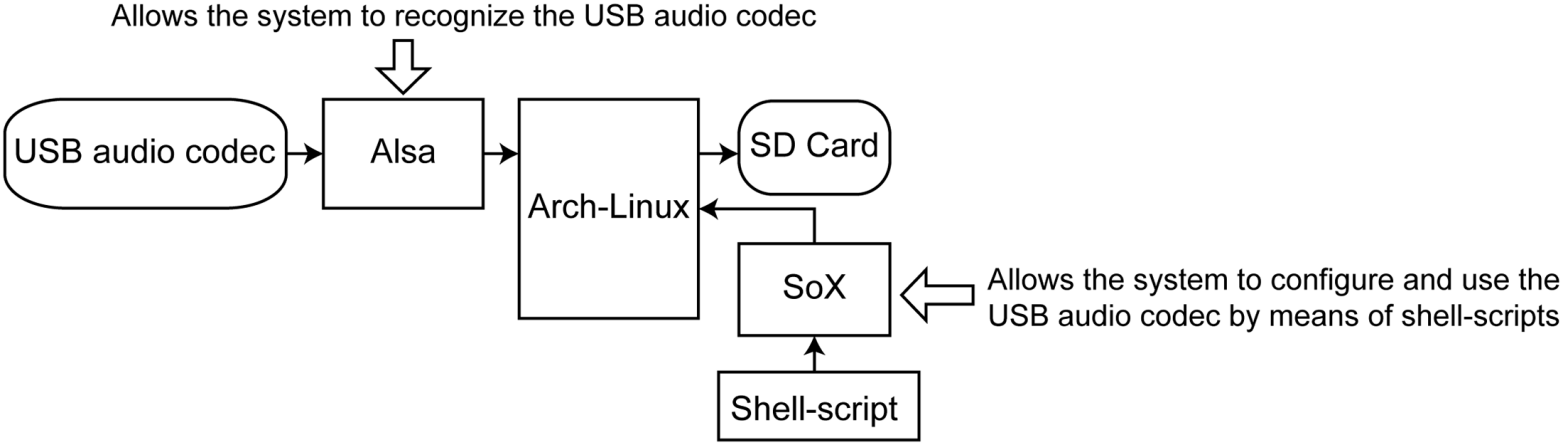
Schematic representation of the software/hardware interaction needed in order to capture the audio signals.

The interaction between the described software and the hardware, necessary in order to correctly store the acoustic data on the SD card, is shown schematically in Fig 3.

All the programming was written and tested using an additional Raspberry Pi Model B, which was accessed via Ethernet. After the programming was done, the SD card was transferred to the Raspberry Pi Model A installed in the device, which executed all the programmed operations equally well. Alternatively, the Raspberry PI Model A could be programmed directly using both a display and a keyboard; however, testing was more time consuming as this model only has one USB port, and had to be swapped between the keyboard and the USB audio codec. In order to retrieve the recorded files, only the SD card was needed. Data could be accessed by any computer capable of reading Linux partitions.

Given that the Raspberry Pi lacks an onboard clock, the time base was provided externally by a Maxim DS3231 real time clock (RTC), which communicates with the board via the GPIO pins 2 and 3 using the I^2^C protocol. This RTC keeps time with an accuracy within ±2 minutes per year while being able to operate from -40°C to +85°C [19].

As storage capacity limits the amount of data that can be collected (and hence affects the autonomy), a 128 GB Kingston SDXC Class 10 card was used in the device. This is one of the largest commercially available SD cards compatible with the Raspberry Pi [20].

### Power management module

**Fig 4:**
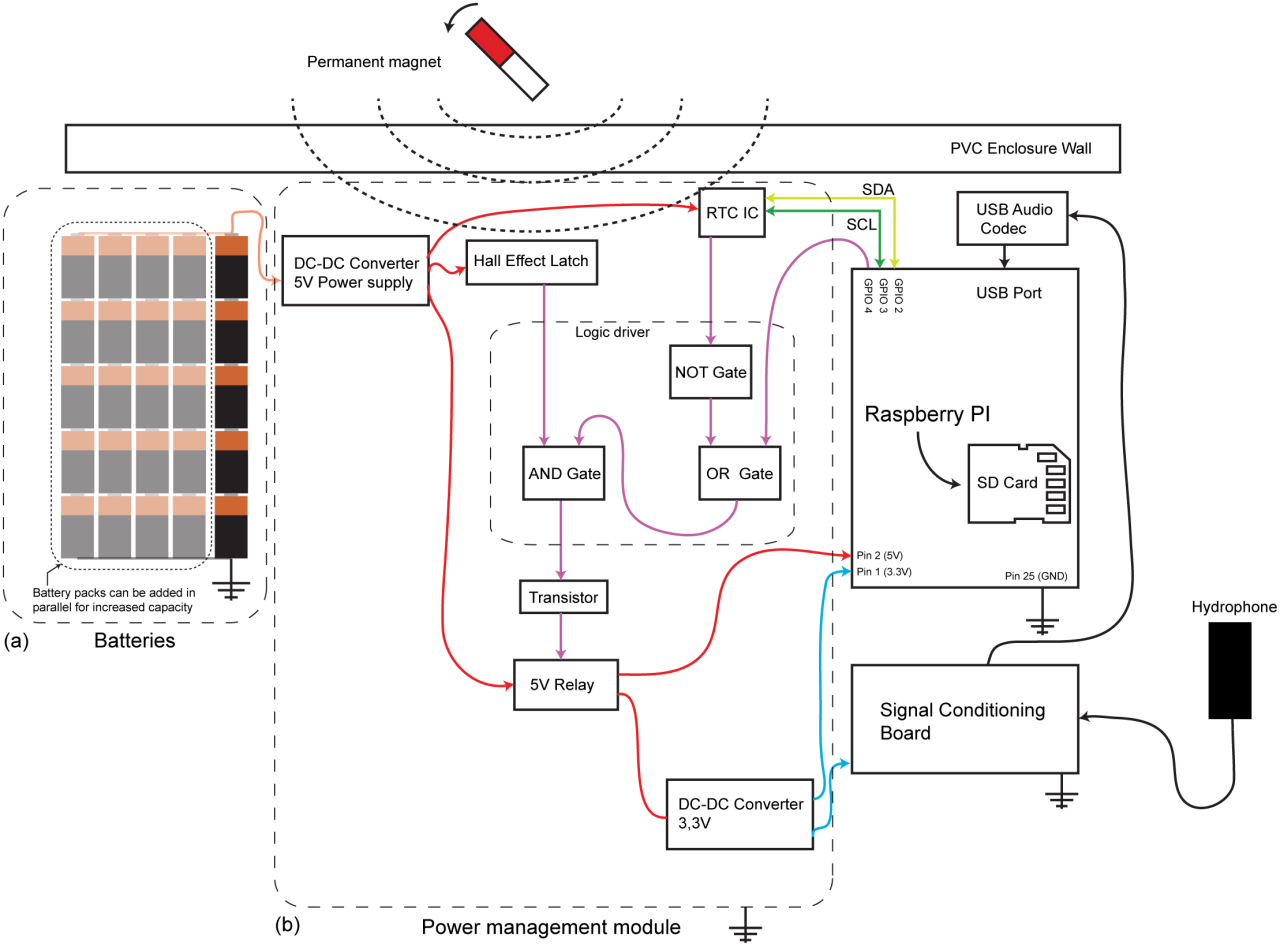
A schematic representation showing the connections made between the components of the underwater recording device.

Other factor that affects the autonomy is the power consumption. During testing, the device was powered by a battery pack formed by five D-size alkaline-manganese dioxide batteries (Duracell MN1300) wired in series. This pack was measured and had a total of 8 V (1.5 V nominal per battery, 1.6 V measured when brand new). Alternatively, as shown in Fig 4a, more packs could be connected in parallel in order to increase the capacity. The output of the battery pack was controlled by a pair of synchronous rectified step-down converters (MP2307) with an efficiency as high as 95% [21]. The first converter was set at 5 V, which is the operating voltage of the Raspberry Pi. The second converter, set at 3.3 V, was a more efficient alternative for the original onboard linear dropout voltage regulator (which was removed from the board).

Since the device was not equipped with neither an external switch nor a communication programming port to be turned-on; energy-wise, it was not convenient to have the device start recording before arriving at the deployment location, which could take hours, days or even weeks. Accordingly, the device was equipped with a Hall effect latch (Allegro A1250) that, under specific conditions, enabled a micro power relay (Omron G6L-1P 5V) to connect or disconnect the battery pack from the electronics. The Hall effect latch was controlled from the exterior of the PVC enclosure using a permanent magnet. When exposed to a south magnetic field, the Hall effect latch produced a logic HIGH output and vice versa.

Nonetheless, in an effort to further increase battery saving, a scheduling feature was implemented using the RTC; which allowed periodic recordings (e.g., during some specific set of hours per day). The RTC, which was directly powered by the 5 V DC-DC converter, is provided with a pin that outputs a digital alarm signal that can be software-programmed (RTC PIN 3). When not programmed or in standby, RTC PIN 3 stays on HIGH state, changing to LOW only when a programmed alarm goes off. This change of state could be used to polarize directly a small transistor serving as a switch to drive the relay. However, for the alarm to function more than once (e.g., an alarm set to be repeated daily), the RTC must be re-programmed each time an alarm is triggered. This was necessary in order to bring RTC PIN 3 to the standby state again. Usually, this can be done by means of a microcontroller, which might not be a straightforward solution for the average user. For the sake of simplicity, a workaround was implemented using a logic driver, which was built using a set of micro power logic gates that enabled the Raspberry Pi to do the re-programming on its own and turn-off the device at some scheduled time. This was achieved by only energizing the relay when certain combinations were input into the logic driver. The whole system is schematically shown in Fig 4.

**Fig 5:**
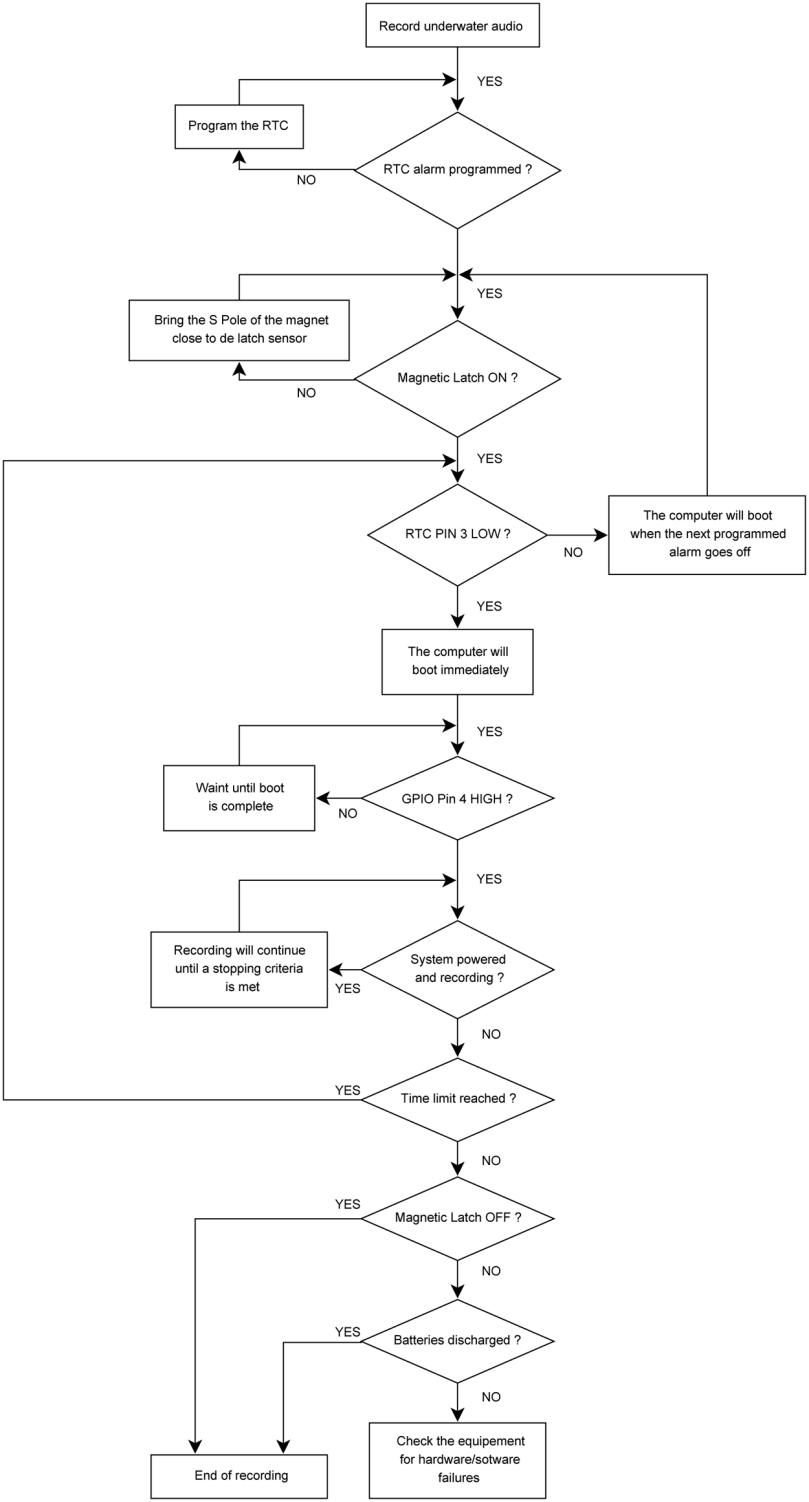
Flow chart of the procedure used to perform scheduled recordings using the resources of the RTC.

The sequence of steps detailed in the flow chart of Fig 5 was used for re-programming the alarm. The combinations for the logic driver are shown in Table 1. The resulting logic state was used to drive the relay, by means of a general purpose NPN transistor (2N2222A).

**Table 1:** Truth table showing the resulting state of the logic driver circuit.

Magnetic Latch	RTC PIN 3	GPIO PIN 4	Relay State
HIGH	HIGH	LOW	DISABLED
HIGH	LOW	LOW	ENABLED
HIGH	LOW	HIGH	ENABLED
HIGH	HIGH	HIGH	ENABLED
LOW	HIGH	LOW	DISABLED
LOW	LOW	LOW	DISABLED

For the procedure to be successful, it was necessary to use a RTC previously programmed to be in standby state (RTC PIN 3 HIGH). First, the magnet was used to activate the Hall Effect latch, which did not energize the relay directly but instead sent a signal to the NOT gate of the logic driver. As soon as certain programmed time condition was met, RTC PIN 3 changed its state to LOW. In sequence (as show in the truth table) the relay became active, powering the Raspberry Pi. After the boot process was completed, a command line instruction was issued in order to change the state of GPIO PIN 4 from LOW to HIGH (only to be turned-off after the designated recording time has elapsed). In sequence, another command line was issued with the purpose of reversing the state of RTC PIN 3 from LOW to HIGH, thus enabling the alarm for the next recording cycle. At this point, the system kept working until the recording time was reached, subsequently switching GPIO PIN 4 from HIGH to LOW in order to deactivate the relay (and accordingly turning the Raspberry Pi off). It remained off (device in sleep state) until the next programmed alarm reverted the state of RTC PIN 3 to LOW, which enabled the relay once more. This way, during a long-term mission (with the magnetic latch continuously in HIGH) the cycle would repeat either until the device is retrieved and manually turned-off or in the event a hardware/software failure (for example, when the batteries go below their cut-off voltage). The logic driver in conjunction with the RTC, the Hall effect latch, the step-down converters and the relay (including its driving transistor) compose the power management module, as illustrated in Fig 4b.

### Power consumption testing

In order to discover any possible influence that the sampling rate might have over the autonomy of the device, a series of tests were run with the intention of monitoring both the instantaneous voltage and the total current drain. Initially, the device was energized with a benchtop power supply set to provide 7.5 V at constant current. Before performing the tests, all non-critical processes that occur during the Raspberry Pi boot cycle (such as video output) were disabled via software. First, with the intention of establishing the minimum power consumption levels, the average power consumption of the device was measured in standby state (i.e. all electronics were powered but not recording) and in sleep state (i.e. all electronics were disabled except for the 5 V DC-DC converter). Successively; 8 hour audio recordings were made, one at a time, using all the available sample rates offered by the USB codec in order to determine the average power consumption for each case. The average power at 8 kHz could not be measured since, at this rate, the recording always occurred at the next nearest value of 11.025 kHz; this might be a limitation either of SoX or the Raspberry Pi. Similar to the case of the USB audio codec, almost any off-the-shelve hand-held audio recording device allows to adjust the sample rate; however, usually offering fewer options to choose from.

However, besides being able to record at various sample rates, the Raspberry Pi has resources that allow for some post-processing to take place as well; for example, filtering or resampling (through the use of SoX commands). This kind of post-processing can be useful because storage space can be saved, as well as offline post-processing time, depending on the bandwidth of the target signals of interest. In order to detect a possible increase in power consumption due to post-processing, the initial 8 hour experiments were replicated using an additional low-pass filtering operation for each case (using a cut-off frequency equal to half-bandwidth of the corresponding sample rate).

Finally, an endurance test was conducted both to determine the characteristic battery discharge behavior of the device and to estimate its autonomy. During this test, the device was powered by a single battery pack. Also, the recording was made at 48 kHz while re-sampling to 24 kHz instead of filtering, as done in the prior test.

All the tests described in this section took place at a temperature of 25°C. All the files were recorded using a single channel (mono) at a 16 bit resolution, formatted as PCM WAV.

### Data management

As the size of collected data increases, it becomes more difficult to analyze. Long-term deployments can result in a huge amount of data. For instance, if the power supply was unlimited, a 128 GB SD card (discounting the 2 GB needed for the operating system) would be able to sore data produced by a continuous mono recording lasting approximately between 15.55 days (48 kHz sample rate) to 67.72 days (11.025 kHz sample rate). Before deploying the device, the RTC is synchronized to the UTC time for the sake of organizing the successive 300 s recordings. They were named following the convention “YYYY.MM.DD_hh.mm.ss,sss.WAV”. A database was consolidated using the MATLAB software in order to manage these files. This database allowed the selection of a portion of audio of interest (based on its initial and final date and time); making it possible to join its composing files as needed and process them as a single file.

Using the calibration constant of the USB audio codec, it was possible to obtain the acoustic pressure waveform and subsequently to calculate parameters such as the SPL relative to 1 μPa. Using the SPL waveforms, it was also possible to calculate statistical parameters, based on window functions, such as the mean or the RMS value. In addition to time based processing, frequency domain calculations (such as the spectrogram) could be performed as well.

### Field deployments

Some autonomous underwater recorders underwent field tests at three different locations along the eastern and southeastern Brazilian coast with the purpose of evaluating their performance as well as the quality of the collected data.

The first deployment site was located in the southeastern coast of Brazil, near the city of Santos (24° 00' 31.1" S 46° 19' 34.8" W). A recorder was installed at a depth of 11 m with the aim of monitoring marine traffic in a public area situated near the entry of the port of Santos. Container-ship transit is steady because it is one of the busiest ports in Latin America. Nevertheless, some smaller ships might be found in the region; for example, outboard powered motorboats which serve as transportation for the local population. The equipment was programmed to record continuously at 22.050 kHz. Later, the sample rate was re-programmed to 16 kHz since this rate was better suited to the sounds being recorded, and it also contributed towards saving storage space.

The second deployment site was located in the eastern coast of Brazil, near the city of Ilhéus (14° 24' 30.35" S 38° 59' 51.87" W). The recorder was installed at a depth of approximately 20 m, with the purpose of registering local populations of marine mammals, particularly of the *Megaptera novaeangliae* species (Humpback whale). These animals are known to migrate from feeding grounds at high latitudes near Antarctica to breeding grounds at lower latitudes close to the Brazilian seaside during the winter [22]. Recordings were scheduled to take place daily between 7am and 5pm. The sample rate chosen for this mission was 11.025 kHz, which was judged adequate given that this species has a dominant frequency range that goes from 120 Hz to 4 kHz [23].

The third deployment site was located in the southeastern coast of Brazil, inside Sepetiba bay (22° 58' 26.4" S 44°00' 00.2" W). The device was suspended in the water column at a depth of 7 m. The recording sampling rate was set to 44.100 kHz as the goal was to detect the presence of members of the *Delphinidae* family.

## Results and Discussion

The results presented in this section provide a good benchmark of the power consumption under typical working conditions; which allow for estimating parameters such as the autonomy, the discharge characteristics of the battery pack and the storage space needed for a given recording scheme. These parameters can be used to optimize the equipment in order to provide the best performance according to the requirements of each specific mission. It was verified that the self-noise of the device was appropriate for recording at the selected field locations. This implies that the sample spectrograms presented at the end of this section are consistent.

### Power consumption and data management

**Fig 6:**
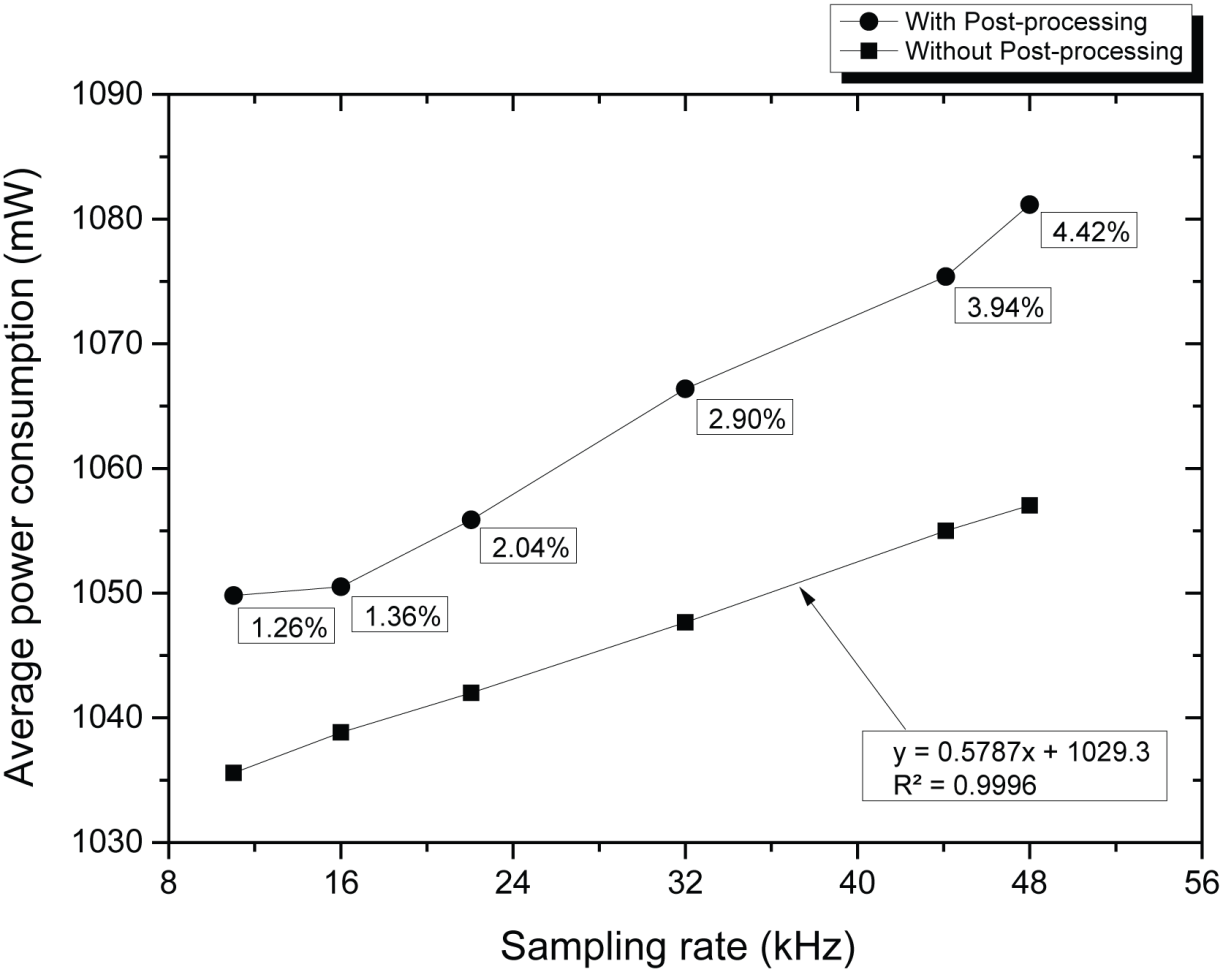
Average power consumption (at constant current) during continuous recordings made at different sample rates, with and without post-processing.

The first parameters measured were related to the absolute minimum power consumption levels. It was observed that in average, the device consumed 1035.70 mW and 89.50 mW (at constant current) while in standby and in sleep respectively. Thus, by using the power management module during non-recording periods, it is estimated that an economy of up to 91.35% could be achieved. This would be crucial for long-term missions. Subsequently, the average power consumption (during recordings corresponding to each sample rate) was calculated. The results are shown on the lower data-set of Fig 6, where it can be noted that power consumption increases linearly with the recording sampling rate.

In contrast, during the second test (which included low-pass filtering) the power consumption increase was non-linear relative to the sample rate. Additionally, it can be observed that power consumption intensified due to the post-processing operation. The percent increase values, related to the corresponding standby state, are shown below the upper data-set of Fig 6.

**Fig 7:**
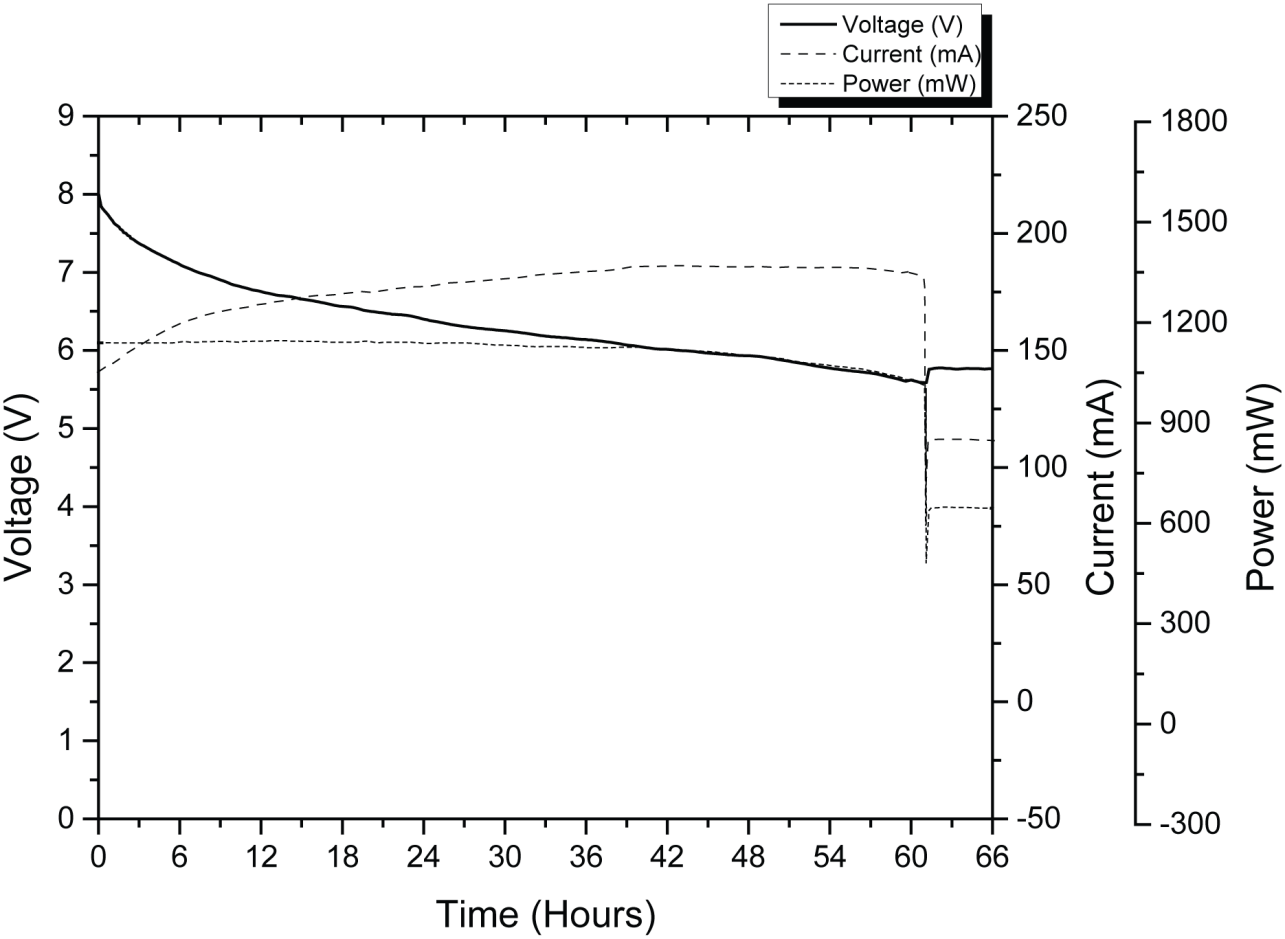
Battery discharge behavior registered while recording (at 48 kHz) and post-processing (resampling).

Subsequently, while performing some experiments in preparation for the third test; it was perceived that whenever the supply voltage was adjusted, the current drain of the device varied as well (keeping the power constant). These observations were coherent with the behavior during the first portion of the endurance test, portrayed in Fig 7. It can be seen that for the first 40 hours (1.66 days) the battery pack discharged at a constant power of about 1150 mW. From this point, and for the next 21 hours (0.88 days), the battery pack began to discharge at an approximately constant current of 185 mA. The device continues to work fine until 61 hours (2.54 days) had elapsed. At that time, the batteries were no longer able to power the device and the Raspberry Pi stopped functioning. This point was highlighted by a dramatic power drop. The cut-off voltage was measured and had a value of 5.58 V (1.12 V per battery). Lastly, by integrating the power over the 61 hours of functioning time, it was found that the total energy delivered by the battery pack was 68.29 W·h; which gave an average power consumption of 1120mW. The integration of current over the same period of time yielded the capacity of the battery pack under the test conditions, which was of 10.79 A·h. However, it was expected that, at lighter working conditions (lower sampling rates, no post-processing, non-continuous recordings) the autonomy could be increased.

Based on the energy delivered by the battery pack during this last test, It is predicted that by lowering the sample rate to 11.025 kHz (1035.71 mW, without post-processing) the autonomy would further increase to 65.93 hours (2.74 days) of continuous recording. By fitting the 5 battery packs allowed by the geometry of the PVC enclosure (as in Fig 4a), the energy capacity would be incremented to 53.95 A·h; bringing autonomy to 329.65 hours (13.70 days) while generating 24.98 GB worth of data.

Still under the last conditions, if scheduling was used instead (e.g. recording 1 hour per day), a good estimate of the expected autonomy could be made using the current drain measured (at constant voltage) both in sleep and standby (which was of 11.93 mA and 138.09 mA respectively). The battery capacity needed for the device to function 1 day is equal to 412.48 mA·h (138.09 mA· 1 hour + 11.93 mA·23 hours); then, 5 battery packs would allow for 130.79 days (approximately 4.29 months) while generating 10.14 GB worth of data. Considering that the operating system needs around 2 GB of space, a 16 GB card would be adequate for this mission.

Indeed, this kind of estimates could be useful to save costs. The predicted recording time could be used to choose smaller SD cards, coherent with the expected mission length.

### USB audio codec calibration and self-noise

**Fig 8:**
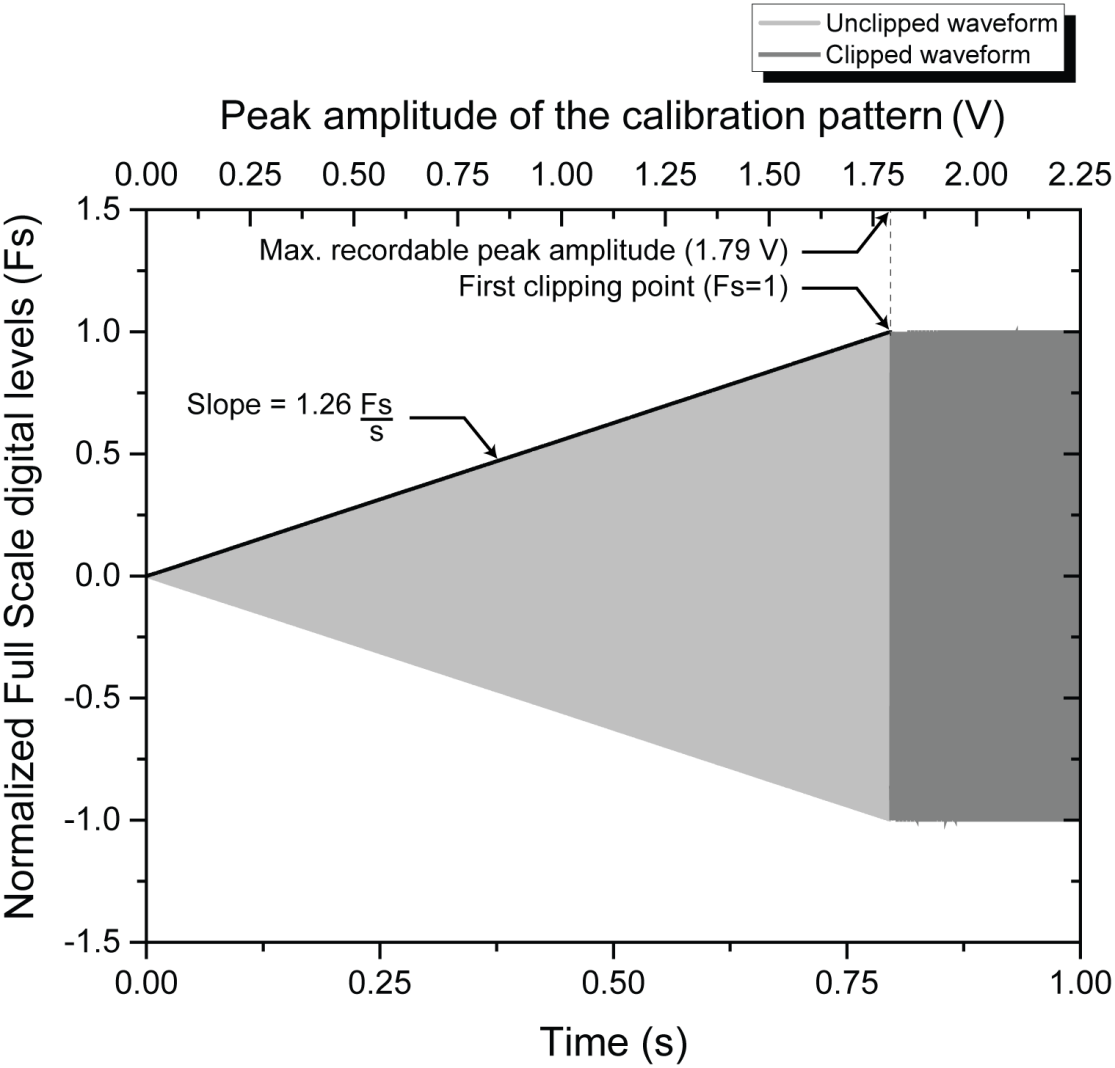
Recorded waveform of the calibration pattern (including clipping).

As it can be seen in Fig 8, the recorded signal (S1 File) corresponded to the shape of the calibration pattern, until it saturated the internal ADC of the USB audio codec. The rate of change of the Fs with respect to time could be calculated outside of the saturated region and had a value of 1.26 Fs/s. As the peak amplitude of the calibration pattern changed at a rate of 2.25 V/s, the calibration constant was calculated and had a value of 1.79 V/Fs. The maximum input amplitude that could be registered before reaching the first clipping point was of 3.58 Vp-p (approximately 2.05 dBV).

Once the calibration constant was found, it was used in conjunction with the voltage sensitivity of the hydrophone in order to measure the lowest noise level of acoustic pressure that could be recorded. Following the procedure described in *Materials and methods*, the self-noise measured was of 84.22 dB_RMS_ re: 1 μPa.

### Audio samples collected during field deployments

**Fig 9:**
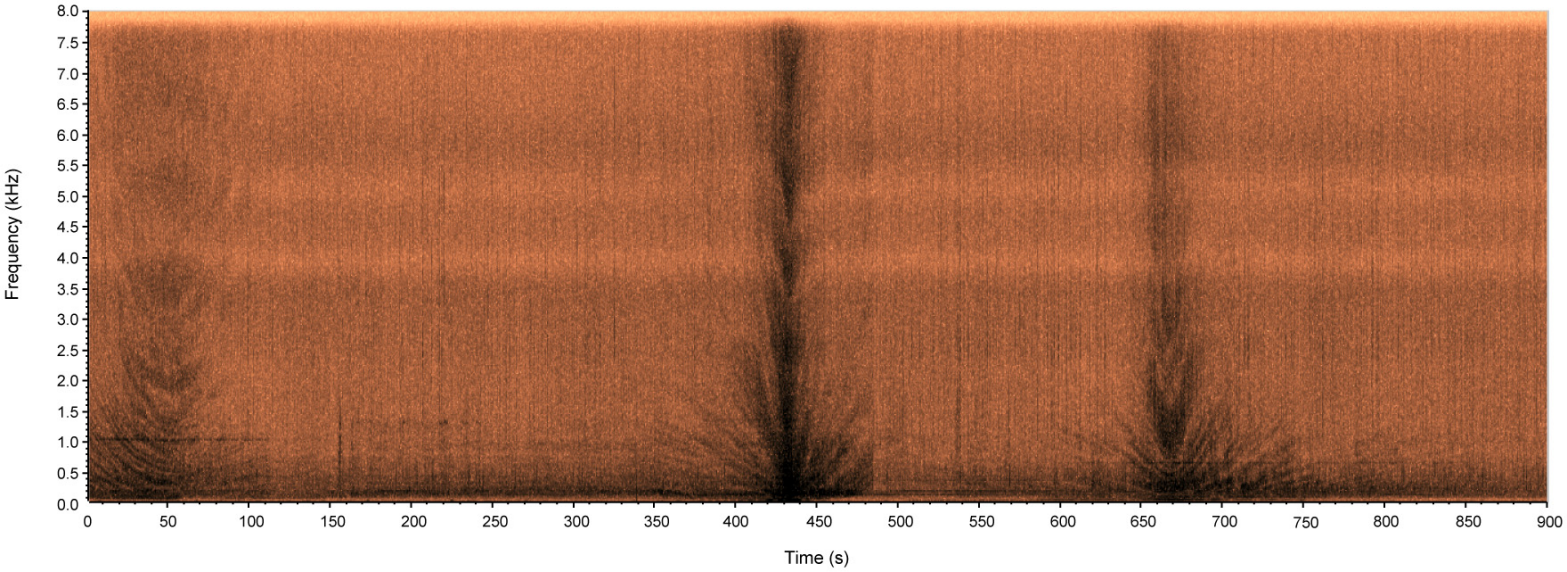
Spectrogram depicting marine traffic near the city of Santos, SP, Brazil.

The image depicted in Fig 9 corresponds to the spectrogram of a portion of audio recorded near the entry of the port of Santos (S2 File), where the measured background noise was of 110.02 dB_RMS_ re: 1 μPa. It mainly represents the sound produced by some small sized boats which seemingly were traveling close to the recorder. In this spectrogram, it can be seen that one of the boats (450 s into the recording) passed faster and that (probably) was closer to the device given that it produced a higher level of intensity (darker regions in the spectrogram). Complementing this kind of measurement with photographic records, would be very useful in order to create a database that could eventually lead to recognize ships by their sound signature.

**Fig 10:**
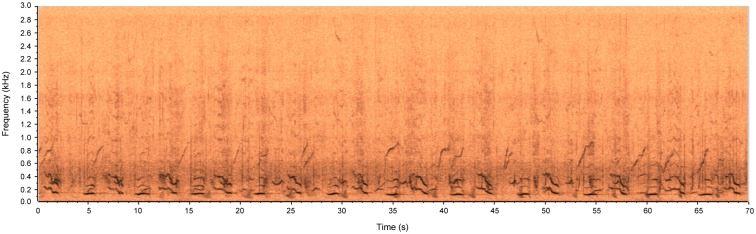
Spectrogram of Humpback whale vocalizations registered in the seaside of the city of Ilhéus, BA, Brazil.

An additional recording unit, installed near the coast of the city of Ilhéus, registered a portion of audio (S3 File) represented by the spectrogram of Fig 10. In this spectrogram, it is possible to identify the periodic components of a certain humpback whale vocalization. The spectrogram was zoomed to the lower 3 kHz; which evidenced that the greater components of those vocalizations were inferior to 500 Hz. At this location, the background noise measured was of 129.18 dB_RMS_ re: 1 μPa.

**Fig 11:**
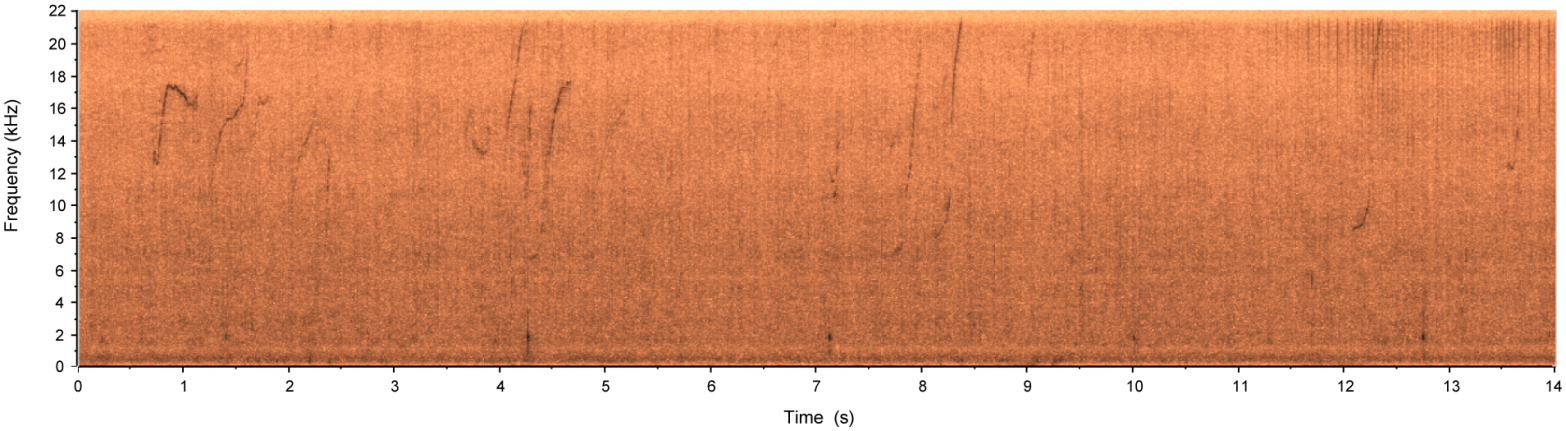
Spectrogram of estuarine dolphin vocalizations registered at Sepetiba Bay, RJ, Brazil.

A third recording unit was installed at Sepetiba bay, where the background noise measured was of 118.52 dB_RMS_ re: 1 μPa. It registered some characteristic whistles that indicated the presence of *Sotalia guianensis* individuals (estuarine dolphins), as it can be detailed in Fig 11 (S4 File). Despite having a vocal range that goes above the 22.050 kHz band that was used, several vocalizations were captured (including the lower frequency portion of some echolocation sounds). This was enough to achieve the objective of detecting their presence.

## Conclusions

This work presented an autonomous underwater recorder based on the Raspberry Pi (Model A) single board computer, which can be assembled and made functional without the need low level programming or specialized hardware, such as microcontrollers or microprocessors. However, some notion of programming is needed, as the user is allowed to perform configuration tasks by means of shell-scripts under a Linux operating system. Those tasks can allow, for example, setting the recording sample rate, scheduling the recordings and post-processing (e.g., resampling and filtering).

Acoustic audio signals were digitalized by means of a PCM2902 USB audio codec connected to the Raspberry Pi. This audio codec proved to be capable of digitalizing signals (having amplitudes as high as 2.05 dBV) with a maximum resolution of 16 bit, at sample rates of up to 48 kHz. The calibration procedure yielded a constant of 1.79 V/Fs.

It was determined that the maximum autonomy achieved using one single battery pack, while recording and post-processing a mono file at 48 kHz, was of 2.54 days. During that time, the battery pack delivered a total energy of 68.29 W·h. The measured capacity of the battery pack until reaching the cut-off voltage of 5.58 V (1.12 V per battery) was of 10.79 A·h. The average power consumed by the device was of 1120 mW. It was estimated that under lighter working conditions, by using the 5 battery packs allowed by geometry of the enclosure, the autonomy for continuous recording would be of 13.70 days; while a scheduled recording scheme of 1 hour per day would increase the functioning time to 4.29 months. Extensive tests of the prototypes showed that a compromise between storage space and long-term functioning can be achieved by means of fine adjustments according to the goal of the mission, as for example, selecting lower sampling rates or programming scheduled recordings.

Apart from the configuration used to test the performance of the device, other configurations might be possible without meaning that they would be less effective. For instance, by further taking advantage of the processing resources of the Raspberry Pi (or any extra resources offered by other suitable single board computer), it would be possible to implement features; such as, recording based on acoustic level detection. As only certain events within frequency and amplitude limits would trigger the recording, this measure would contribute with power saving and would also lower the amount of data to be stored and processed. Other configuration variations can include (but are not limited to), for example, another kind of hydrophone (with different sensitivity, dimensions, shape or directional pattern) or a different material for the enclosure (which could modify the depth rating).

The device proved to be useful for acquiring, processing and storing signals from biological and anthropogenic events at selected locations along the eastern and southeastern coast of Brazil. All field recordings were made with a hydrophone with a sensitivity of -198 dB re: 1V/μPa in conjunction with a 48 dB pre-amplifier. The self-noise with this configuration was of 84.22 dB_RMS_ re: 1 μPa, which was lower than the background noise values measured at the deployments sites.

By altering the recording parameters (or by adding post-processing), the user can easily adapt the equipment accordingly to the nature of each mission. The cost of replication of the device was estimated to be around 500 USD. Given that basic hydrophone amplifiers, such as [24], are in the range of 500 USD [25] and that specialized underwater autonomous recorders have prices close to 6000 USD [4]; the device proposed in this work offers a good value for the money and provides an excellent low-cost alternative for research related activities.

## Supporting Information

S1 File Audio file containing a recording of the calibration pattern, used to produce Fig 8. The saturation of the internal ADC of the USB audio codec can be observed when opening the file in any suitable software capable of identifying clipping.
(WAV)

S2 File Audio file containing a recording of the audio represented by the spectrogram shown in Fig 9. (WAV)

S3 File Audio file containing a recording of the audio represented by the spectrogram shown in Fig 10. (WAV)

S4 File Audio file containing a recording of the audio represented by the spectrogram shown in Fig 11. (WAV)
